# A genome-wide BAC-end sequence survey provides first insights into sweetpotato (*Ipomoea batatas* (L.) Lam.) genome composition

**DOI:** 10.1186/s12864-016-3302-1

**Published:** 2016-11-21

**Authors:** Zengzhi Si, Bing Du, Jinxi Huo, Shaozhen He, Qingchang Liu, Hong Zhai

**Affiliations:** Beijing Key Laboratory of Crop Genetic Improvement/Laboratory of Crop Heterosis and Utilization, Ministry of Education, China Agricultural University, Beijing, 100193 China

**Keywords:** Sweetpotato, BAC library, BAC-end sequence, SSRs, Comparative genome

## Abstract

**Background:**

Sweetpotato, *Ipomoea batatas* (L.) Lam., is an important food crop widely grown in the world. However, little is known about the genome of this species because it is a highly heterozygous hexaploid. Gaining a more in-depth knowledge of sweetpotato genome is therefore necessary and imperative. In this study, the first bacterial artificial chromosome (BAC) library of sweetpotato was constructed. Clones from the BAC library were end-sequenced and analyzed to provide genome-wide information about this species.

**Results:**

The BAC library contained 240,384 clones with an average insert size of 101 kb and had a 7.93–10.82 × coverage of the genome, and the probability of isolating any single-copy DNA sequence from the library was more than 99%. Both ends of 8310 BAC clones randomly selected from the library were sequenced to generate 11,542 high-quality BAC-end sequences (BESs), with an accumulative length of 7,595,261 bp and an average length of 658 bp. Analysis of the BESs revealed that 12.17% of the sweetpotato genome were known repetitive DNA, including 7.37% long terminal repeat (LTR) retrotransposons, 1.15% Non-LTR retrotransposons and 1.42% Class II DNA transposons etc., 18.31% of the genome were identified as sweetpotato-unique repetitive DNA and 10.00% of the genome were predicted to be coding regions. In total, 3,846 simple sequences repeats (SSRs) were identified, with a density of one SSR per 1.93 kb, from which 288 SSRs primers were designed and tested for length polymorphism using 20 sweetpotato accessions, 173 (60.07%) of them produced polymorphic bands. Sweetpotato BESs had significant hits to the genome sequences of *I. trifida* and more matches to the whole-genome sequences of *Solanum lycopersicum* than those of *Vitis vinifera*, *Theobroma cacao* and *Arabidopsis thaliana*.

**Conclusions:**

The first BAC library for sweetpotato has been successfully constructed. The high quality BESs provide first insights into sweetpotato genome composition, and have significant hits to the genome sequences of *I. trifida* and more matches to the whole-genome sequences of *Solanum lycopersicum*. These resources as a robust platform will be used in high-resolution mapping, gene cloning, assembly of genome sequences, comparative genomics and evolution for sweetpotato.

**Electronic supplementary material:**

The online version of this article (doi:10.1186/s12864-016-3302-1) contains supplementary material, which is available to authorized users.

## Background

Sweetpotato, *Ipomoea batatas* (L.) Lam., is an important food crop widely grown in the world. More than 104 million tons are produced globally, 95% of which are grown in developing countries [1]. It is also an alternative source of bioenergy as a raw material for fuel production [2]. The orange-fleshed sweetpotato is rich in *beta*-carotene, which plays a crucial role in preventing vitamin A deficiency-related blindness and maternal mortality [3]. In addition, polyphenols in sweetpotato leaves are found to suppress the growth of human cancer cells [4]. Sweetpotato is a highly heterozygous and self-incompatible autohexaploid (2n = 6× = 90) and little is known about its genome [5].

Recently, de novo whole-genome sequencing of the selfed line Mx23Hm and the highly heterozygous line 0431–1 of *I. trifida* (2n = 2× = 30, the likely diploid ancestor of sweetpotato) was performed using the Illumina HiSeq platform, but their assembly and annotation are still in the early stages [6]. Transcriptome sequencing of sweetpotato provided an important transcriptional data source for studying storage root formation, flower development and carotenoid and anthocyanin biosynthesis of this species [7–13]. Lang et al. [14] reported the complete nucleotide sequence of the chloroplast genome of sweetpotato using the next-generation sequencing technology. However, the full genomic sequence of the cultivated sweetpotato is still absent.

Bacterial artificial chromosome (BAC) libraries are valuable resources for genome sequencing, physical mapping, analysis of gene structure and function and comparative genomics, in particular for the species unsequenced or of complex genome structure [15–18]. Sequencing the ends of BAC clones is an efficient strategy to produce low-pass genomic sequences, which are used to estimate genome properties such as genome organization and composition, to identify macro- and micro-synteny between species, to facilitate the assembly of contigs into scaffolds during whole genome sequencing and to develop molecular markers [19–22]. BAC-end sequences (BESs) analyses have been conducted in number of plant species such as rice [15], maize [23], wheat [24], apple [25], *Spartina maritima* [26], sugarcane [27], coffee [28] and passion fruit [29]. To date, the BAC library of sweetpotato has not been constructed.

In the present study, the first BAC library of sweetpotato was constructed. Clones from the BAC library were end-sequenced and analyzed to provide genome-wide information about sweetpotato. The analyses focused on GC content, repeat element composition, protein encoding regions, simple sequence repeat (SSR) and genome comparison between sweetpotato and other plants.

## Results

### BAC library construction and characterization

**Fig. 1:**
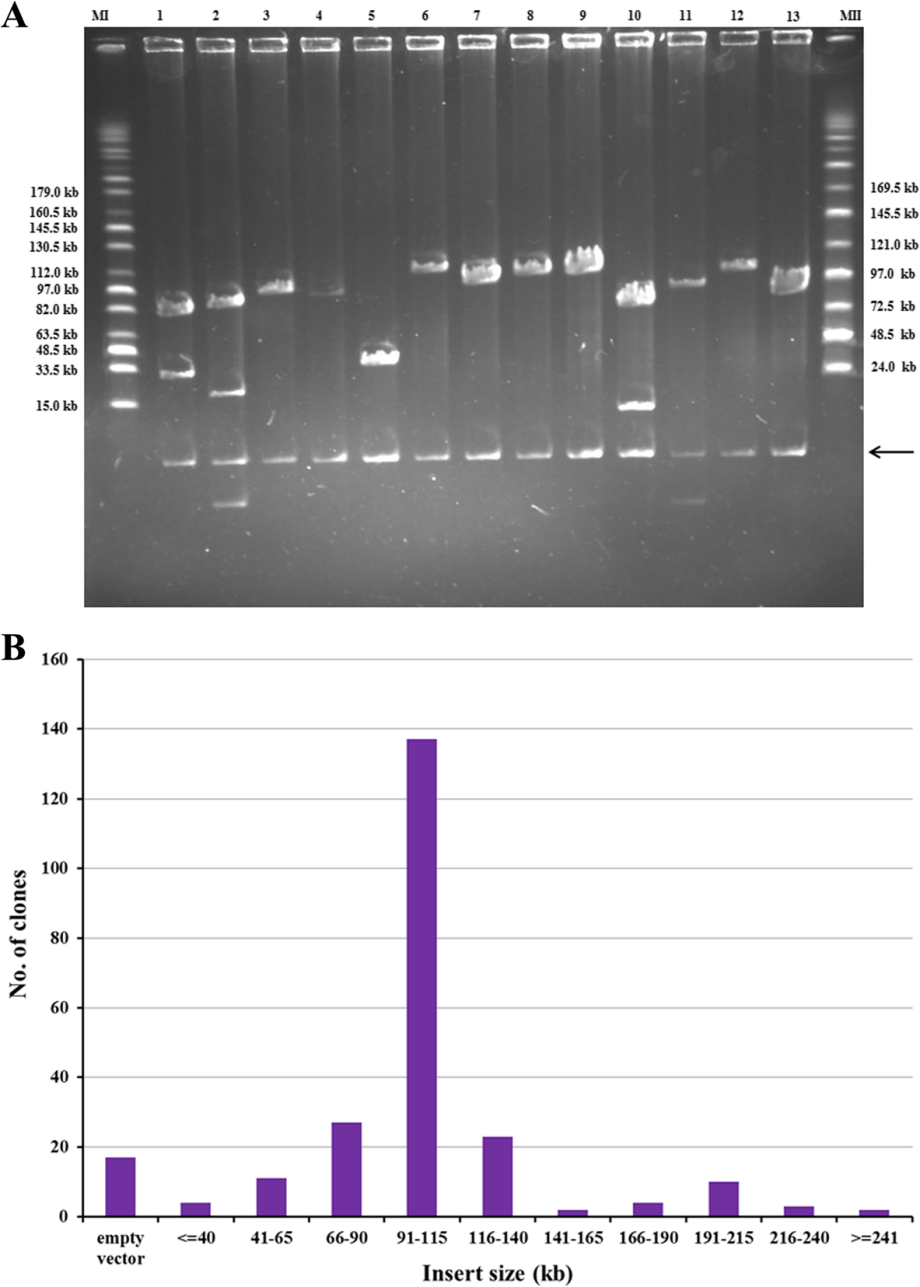
Insert size analysis of the sweetpotato BAC library. **a** Pulsed filed gel electrophoresis of 13 BAC clones DNA digested with *Not* I (Lanes 1–13). MI was MidRange I PFG Marker; MII was MidRange II PFG Marker. The arrow shows the 8.1 kb band of the cloning vector. **b** Distribution of the insert size of 240 randomly selected sweetpotato BAC clones

A sweetpotato BAC library was constructed using sweetpotato line Xu 781 with high dry-matter content and stem nematode resistance by partial digestion of its nuclear DNA with *Hind*III. The BAC library consisted of 240,384 BAC clones stored in 626 (384-wells) microtitre plates, from which 240 clones were randomly selected to estimate the average insert size. The insert size ranged from 15 to 305 kb, with an average size of 101 kb. The majority (75.42%) of the clones had insert sizes of > 90 kb, 15.83% between 40 kb and 90 kb, 1.67% < 40 kb and 7.08% no insert (Fig. 1). Based on the sweetpotato genome size of 2200–3000 Mb [5], the BAC library had a 7.93–10.82 × coverage of the genome, and the probability of isolating any single-copy DNA sequence from the library was more than 99%.

A subset of the library consisting of 1,152 individual BAC clones was screened using mitochondrial and chloroplast gene specific primers. The results indicated that the library had a very low frequency of clones derived from the mitochondrial genome (0.26%) and chloroplast genome (0.43%).

To check the utility of the library in gene isolation, the library was screened with special primers complementary to the sequences of two sweetpotato genes, *IbPPOS* (GenBank: AY822711.1) and *IbMIPS1* [30]. It was found that 14 and 33 clones harbored *IbPPOS* and *IbMIPS1*, respectively, showing the availability of the BAC library for gene isolation.

### BAC-end sequences

**Fig. 2:**
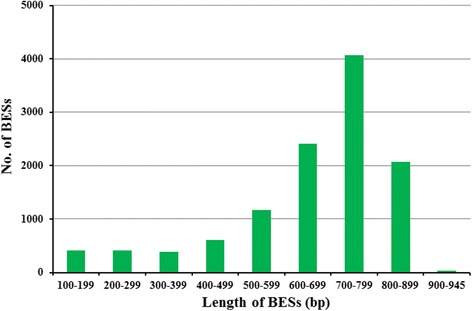
Sizes distribution of the sweetpotato BESs

A total of 8,310 clones were sequenced from both forward and reverse directions and 16,620 raw data reads were produced (Table 1). After trimming BESs for vector and low read quality sequences, 11,598 BESs with a quality score of ≥20 and a sequence length ≥100 bp were obtained. An additional set of 56 BESs was filtered out due to high similarity to *Arabidopsis* mitochondria (GenBank: NC_001284.2) or chloroplast (GenBank: NC_000932.1). The remaining 11,542 BESs, including 9,894 paired-end reads and 1,648 unpaired reads, represented about 7.6 Mb (~0.30%) of the sweetpotato genome and their lengths ranged from 100 bp to 945 bp, with an average length of 658 bp. In terms of length distribution, 700–799 bp were the most abundant categories, accounting for 35.18% (4,061) of all BESs, followed by 600–699 bp (20.82%, 2,403) and 800–899 bp (17.91%, 2,067) (Fig. 2). The GC content was 38.18%, indicating that the sweetpotato genome is AT-rich (Table 1). All BESs of length ≥100 bp were deposited to the GenBank GSS database (accession numbers KS309164–KS320705).

**Table 1:** Summary of BAC-end sequencing

Total number of BESs	16,620
No. of BESs with organellar DNA	56
No. of good quality BESs (Phred quality score >20, read length > 100 bp)	11,542
No. of paired BESs	9,894
No. of non-paired BESs	1,648
Total length of BESs (bp)	7,595,261
Minimum length of BESs (bp)	100
Maximum length of BESs (bp)	945
Average length of BESs (bp)	658.10
GC content (%)	38.18
No. of BESs with repetitive DNA	7,114
No. of BESs with protein coding region	2,088

### Repetitive DNA content and composition

Based on similarity searches in the repeat database, 12.17% (924,646 bp) of the nucleotides in the sweetpotato BESs were identified as known repetitive DNA elements (Table 2). Class I retrotransposons were the most abundant repeats, representing 69.92% of the total repetitive DNA content and 8.51% of the total genomic sequence. They were subdivided into LTR retrotransposons and Non-LTR retrotransposons. LTR retrotransposons, included Ty1-Copia elements and Ty3-Gypsy elements, accounted for 86.52% of the total retrotransposons and 7.37% of the genomic sequence. The number of Ty3-gypsy (1,008) was slightly higher than that of Ty1-copia (873). Non-LTR retrotransposons, including short interspersed elements (SINEs) and long interspersed elements (LINEs), represented 13.48% of the total retrotransposons and 1.15% of the genomic sequence. The number of LINEs (415) exceeded that of SINEs (15). A total of 673 Class II DNA transposons were predicted, representing 11.63% of the total repetitive DNA content and 1.42% of the total genomic sequence. The hobo-Activator was found to be the most elements (168), followed by CMC-EnSpm (133), MULE-MuDR (116) and RC/Helitro (102). In addition, simple repeats, low complexity, small RNA and satellite elements were also identified (Table 2).

**Table 2:** Classification and distribution of repetitive DNA elements in BESs

Class	Number	Length occupied (bp)	Percentage of the genome
Class I retrotransposon	2,414	646,519	8.51
LTR retrotransposons	1,984	559,396	7.37
Ty1/copia	873	276,307	3.64
Gypsy/DIRS	1,008	274,869	3.62
Others	103	8,220	0.11
Non-LTR retrotransposons	430	87,123	1.15
SINE	15	1,018	0.01
LINE	415	86,105	1.13
Class II DNA transposons	673	107,599	1.42
hobo-Activator	168	26,272	0.35
TcMar	26	2,226	0.03
CMC-EnSpm	133	19,577	0.26
MULE-MuDR	116	20,521	0.27
RC/Helitron	102	29,024	0.38
PiggyBac	1	85	0.00
Tourist/Harbinger	24	2,340	0.03
Others	103	7,554	0.10
Unclassfied	11	752	0.01
Total interspersed repeats	3,098	754,870	9.94
Small RNA	34	4,925	0.06
Satellites	26	2,954	0.04
Simple repeats	3,408	147,232	1.94
Low complexity	550	26,125	0.34
Total repetitive DNA	7,116	924,646	12.17

A total of 242 repeat families were detected by RepeatModeler and the lengths ranged from 51 to 889 bp. Ten of them were eliminated due to containing hits to transposon related proteins. The remaining 232 families were not found in the public repeat database and considered as sweetpotato novel repetitive elements, 49 of which were classified as DNA transposons, 92 as LTRs, 28 as LINEs, 10 as SINEs and 53 as unknown. These sweetpotato unique repeats were used as a custom library for RepeatMasker and masked a total of 1,390,919 bp, equivalent to 18.31% of the total genomic sequence. Together with the known repeats, the total repetitive DNA content in sweetpotato genome was about 30.48%.

### Protein coding regions and functional annotation

**Fig. 3:**
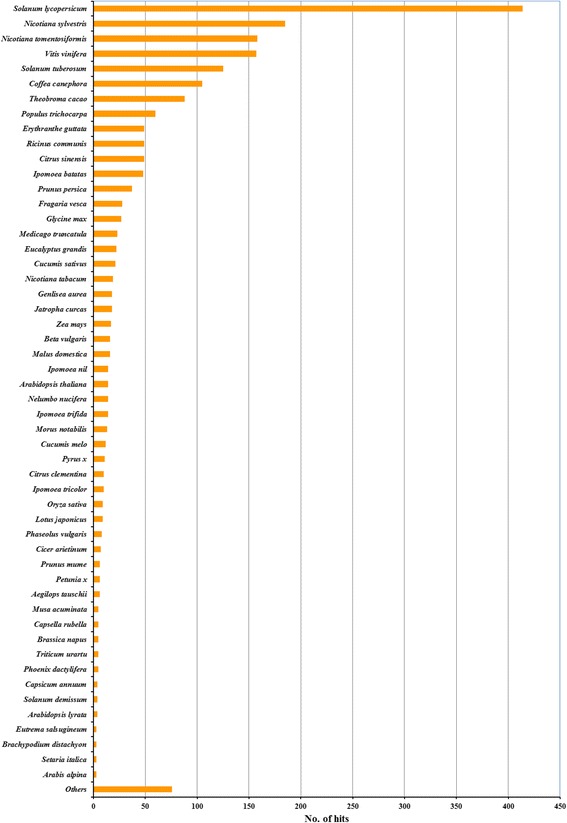
List of plant species that had significant hits to the sweetpotato BESs

After masking the known and novel repeats, the remaining 4,428 BESs were used to identify protein coding regions. A total of 3,360 BESs were found to be homologous to the sweetpotato express sequence tags (ESTs) downloaded from NCBI GenBank and derived from our in-house transcriptome data (unpublished), accounting for 16.43% (1,248,033 bp) of the total length of sweetpotato BESs (Additional file 1: Table S1); 1,526 BESs were of significant hits to NCBI-ESTs database, accounting for 4.77% (362,031 bp) of sweetpotato BESs (Additional file 2: Table S2). Taken together, 3,422 BESs were homologous to ESTs databases, with a cumulative match length of 1,270,851 bp, representing 16.73% of the total BESs dataset (Additional file 3: Table S3). Of these, 2,088 BESs were also of significant hits to NCBI NR database and the cumulative match length was 760,248 bp, accounting for 10.00% of the total BESs dataset (Additional file 4: Table S4). The majority of the top-hits were to *Solanum lycopersicum* (414 BESs), *Nicotiana sylvestris* (185 BESs), *N. tomentosiformis* (158 BESs), *Vitis vinifera* (157 BESs), *S. tuberosum* (125 BESs), *Coffea canephora* (105 BESs) and *Theobroma cacao* (88 BESs) (Fig. 3).

**Fig. 4:**
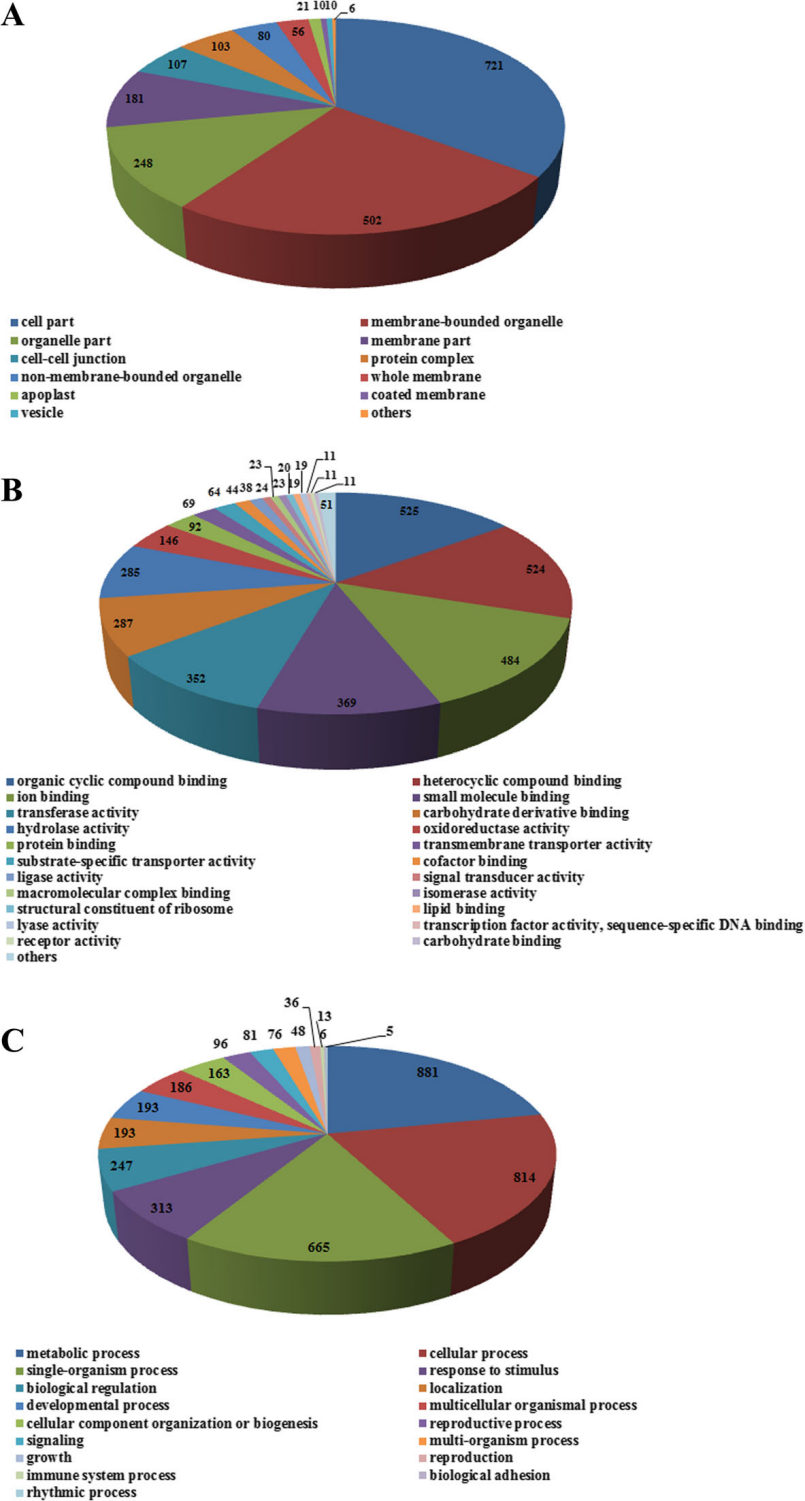
GO classification of the predicted protein-coding genes from the sweetpotato BESs. **a** Cellular components. **b** Molecular functions. **c** Biological processes

Functional annotation showed that 6,790 ontological terms were assigned to 1,526 BESs. These BESs were further classified into three categories: cellular components (866), molecular functions (1,185) and biological processes (919) (Fig. 4). Of the BESs in the cellular components category, 721 (83.26%) were for cell part, 502 (57.97%) for membrane-bounded organelle and 248 (83.26%) for organelle part. The BESs in molecular functions category were distributed as follows: organic cyclic compound binding (525, 44.30%), heterocyclic compound binding (524, 44.22%), ion binding (484, 40.84%), small molecule binding (369, 31.14%), transferase activity (352, 29.70%), carbohydrate derivative binding (287, 24.22%) and hydrolase activity (285, 24.05%). The most represented biological processes were metabolic process (881, 95.87%), hydrolase activity (814, 88.57%) and single-organism process (665, 72.36%).

A total of 2,210 BESs had significant matches to the protein database of *S. lycopersicum*, with a cumulative match length of 773,346 bp, representing 10.18% of the total BESs dataset (Additional file 5: Table S5). Based on an estimated sweetpotato genome size of 2200–3000 Mb, the total coding region length of the sweetpotato genome was predicted to 217.78–296.97 Mb. If the average coding region length in sweetpotato was 1,379 bp, as in *S. lycopersicum*, the total gene content of the sweetpotato genome might be 157,926–215,351. As compared to the *V. vinifera* protein database, 2,127 BESs showed significant matches, accounting for 9.87% (749,931 bp) of the total BESs dataset (Additional file 6: Table S6). The total coding region length of sweetpotato was estimated as 217.14–296.10 Mb and the number of genes was predicted as 146,792–199,932, assuming that an average coding region length was 1,481 bp as in *V. vinifera*. A total of 2,764 and 2,638 BESs showed significant hits to the CDS databases of Mx23Hm and 0431–1 of *I. trifida* (2×), representing 10.71% (813,709 bp) and 11.23% (852,756 bp) of the total BESs dataset, respectively (Additional file 7: Table S7 and Additional file 8: Table S8). The coding regions in sweetpotato were estimated as 235.70–321.40 Mb and 247.00–336.82 Mb and the number of genes was predicted as 153,446–209,245 and 200,816–273,840, according to a gene CDS length of 1,536 bp in Mx23Hm and 1,230 bp in 0431–1, respectively.

### Simple sequence repeats (SSRs)

**Fig. 5:**
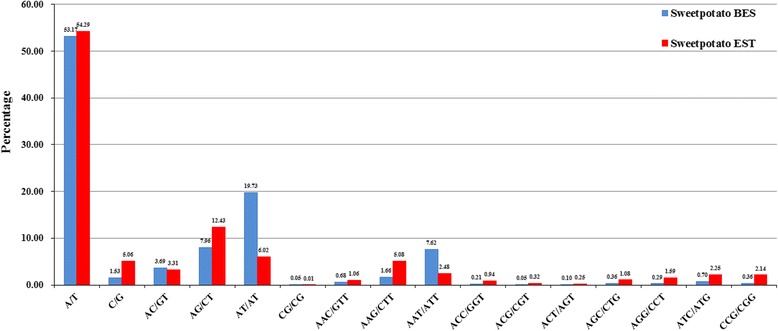
Distribution of SSR motifs in the sweetpotato BESs and ESTs

The 11,542 BESs were subjected to a search for SSRs and a total of 3,846 SSRs were identified from 2,698 BESs. The average density of SSRs in sweetpotato BESs was one SSR per 1.93 kb. Most of SSRs were mono- (54.71%), di- (31.44%) and trinucleotide repeats (12.04%), with less abundant tetra- (1.35%), penta- (0.31%) and hexanucleotide repeats (0.16%) (Additional file 9: Table S9). A/T motifs were the most common mononucleotide repeats, while G/C motifs were present at a much lower frequency (Fig. 5). The most frequently occurring motifs were AT/AT in the dinucleotide repeats and AAT/ATT in the trinucleotides repeats. Thus, AT-rich SSRs were consistently more abundant than GC-rich SSRs. Of the 3,846 SSRs, 3,161 were perfect SSRs containing a single repeat motif, and 685 were compound SSRs composed of two or more SSRs separated by ≤ 100 bp. The perfect SSRs were further subdivided into Class I (≥20 bp in length, 34.55%) and Class II (10–19 bp in length, 65.45%) according to the method of Temnykh et al. [31]. Di- (53.70%) and trinucleotide motifs (20.79%) were of most abundance in class I SSRs, while mononucleotide motifs (66.46%) were most frequently occurred in class II SSRs.

The distribution and frequency of different types of SSRs in sweetpotato BESs were compared with those in the ESTs (Fig. 5). A total of 45,649 EST-SSRs were identified from the sweetpotato ESTs (166,866,793 bp) downloaded from NCBI GenBank and derived from our in-house transcriptome data (unpublished) using MicroSAtellite (MISA). The mononucleotide repeats were the most abundant type (59.35%), followed by di- (21.76%), tri- (17.19%), tetra- (1.40%), penta- (0.23%) and hexanucleotide repeats (0.08%), showing a consistent trend with those in BES-SSRs. Compared to BES-SSRs, the most common motifs were also A/T in mononucleotide EST-SSRs, but the most frequently occurring motifs were AG/CT in the dinucleotide repeats and AAG/CTT in the trinucleotides repeats (Fig. 5). Furthermore, the average density of SSRs in the ESTs was one SSR per 3.66 kb, which was approximately a half of BESs (one SSR per 1.93 kb), indicating that potential SSRs are more numerous in BESs than in ESTs.

**Fig. 6:**
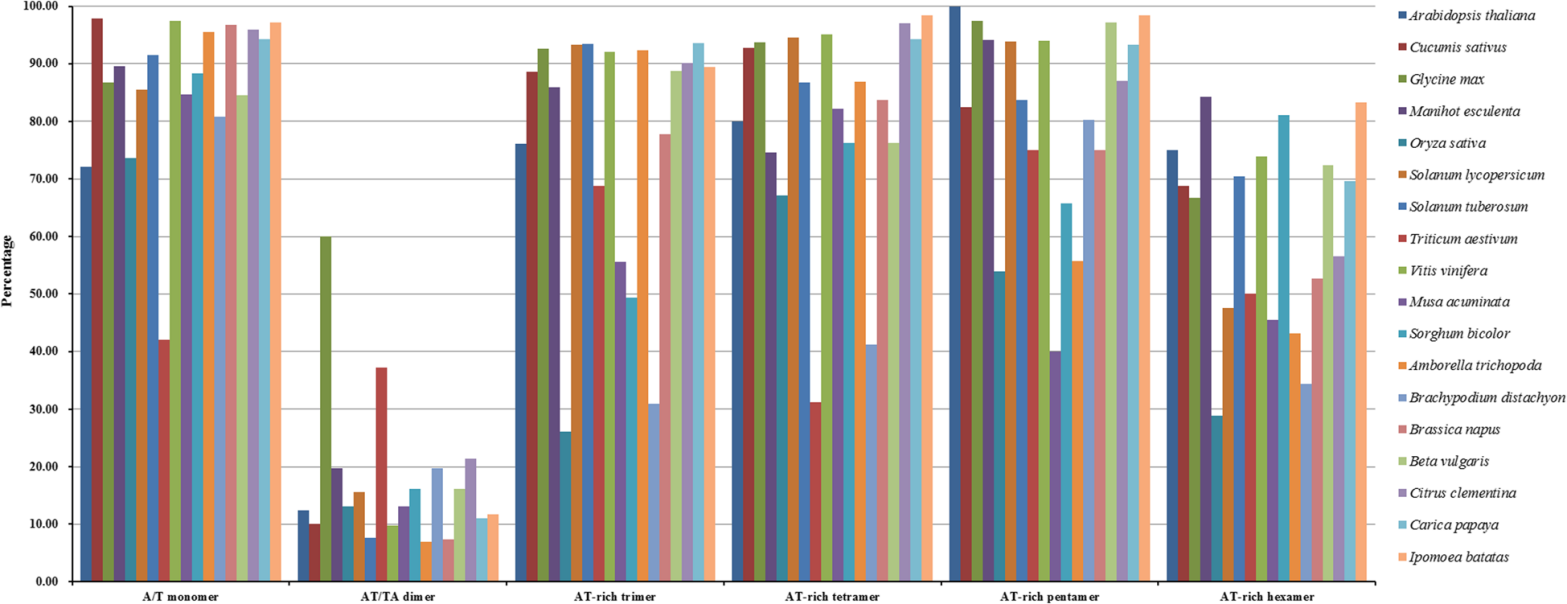
Frequency of AT-rich repeat motifs in BESs of sweetpotato and other plant species

For comparison purposes, analyses were performed to identify BES-SSRs of other species. A consistent trend was found that the proportion of the corresponding SSRs decreased as the length of motif unit increased in most of the surveyed species. Mono-, di- and trinucleotide repeats were dominant in all of the surveyed species, accounting for more than 97% of the total SSRs (Additional file 9: Table S9). The AT-rich SSRs frequently occurred in all of the surveyed species (Fig. 6). In addition, sweetpotato showed a higher density of SSRs among the surveyed species (Additional file 9: Table S9).

Of the 3,846 SSRs, 288 were chosen to design primers for assessing their allelic polymorphisms with 20 sweetpotato accessions (Additional file 10: Table S10.). The 248 primer pairs (86.11%) successfully amplified products from at least 1 of the 20 tested accessions and 173 primer pairs (60.07%) produced polymorphic bands among the 20 tested accessions on denaturing polyacrylamide gels (Additional file 11: Figure S1A). Furthermore, the 173 polymorphic primer pairs were assessed for their allelic polymorphisms with the 20 sweetpotato accessions and 109 of them (37.85%) produced polymorphic bands on agarose gels (Additional file 11: Figure S1B). Twelve of the 109 primer pairs were chosen to amplify 168 F_1_ individuals derived from a cross between Xushu 18 and Xu 781 and 11 of them generated polymorphic bands on agarose gels (Additional file 12: Figure S2).

### Comparative genome analysis

The 11,542 sweetpotato BESs were compared to the genome sequences of Mx23Hm and 0431–1 of *I. trifida* (2×). A total of 11,229 (97.29%) and 11,320 (98.08%) BESs had significant hits to the genome sequences of Mx23Hm and 0431–1, respectively. The matches were scattered in 4,658 contigs of Mx23Hm and 6,738 contigs of 0431–1, with a cumulative match length of 6,229,456 bp (82.02% of the total BESs length) and 6,154,441 bp (81.03%), respectively. Of these BESs, 689 and 210 paired BESs were aligned on the same contigs of Mx23Hm and 0431–1, respectively, in the correct orientation within 15–350 kb apart (Additional file 13: Table S11 and Additional file 14: Table S12). These results support that sweetpotato has a highly close relationship with *I. trifida* (2×).

These BESs were also compared to the sequenced *S. lycopersicum*, *V. vinifera*, *T. cacao* and *A. thaliana* genomes to identify microsyntenic regions. *N. sylvestris*, *N. tomentosiformis*, *S. tuberosum* and *C. canephora* genomes are still in the early stages of their assembly and annotation and are not suitable for comparative mapping studies [29] though they had the high number of top hits to sweetpotato.

**Fig. 7:**
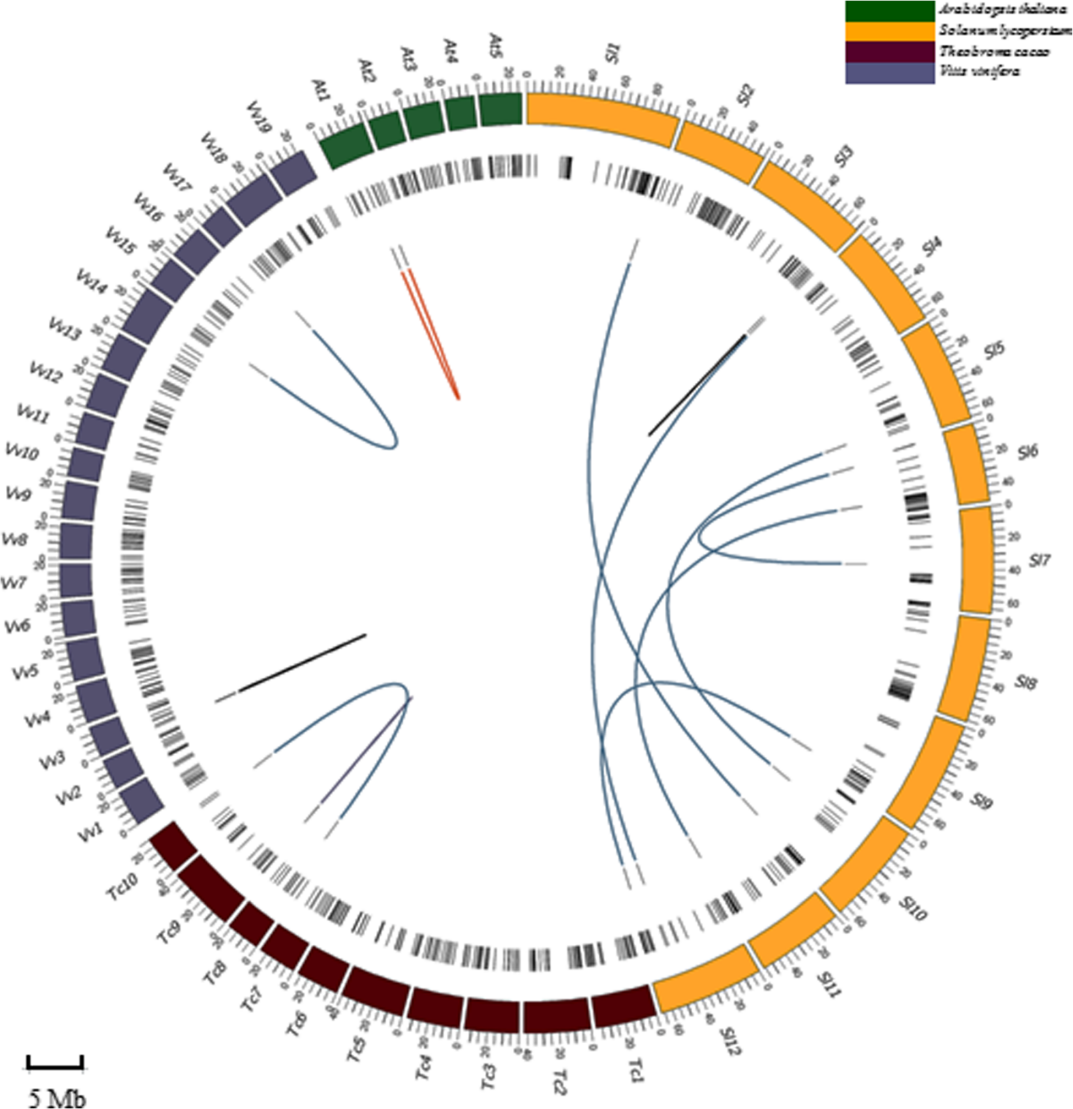
Comparative analysis of the sweetpotato BESs with the genomes of four sequenced plant species. The out circle represents the haploid chromosomes of the four species: *S. lycopersicum (Sl), V. vinifera (Vv), T. cacao (Tc)* and *A. thaliana (At)*; the middle circle represents the matches of single BESs with the four plant genomes; the inn circle represents matches of the paired BESs with the four plant genomes. Paired BESs are linked to each other with links: ‘non-colocalized’ (blue), ‘collinear’ (black), ‘rearranged’ (purple) and ‘gapped’ (red)

**Fig. 8:**
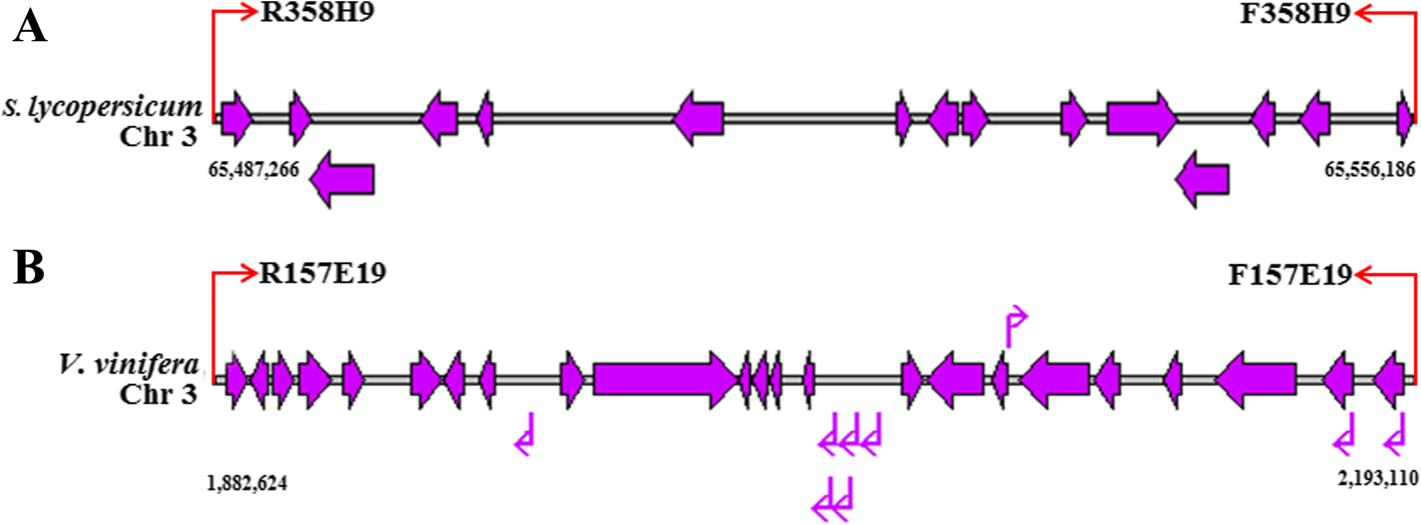
Analysis of microsyntenic regions between sweetpotato BACs and *S. lycopersicum* (**a**) and *V. vinifera* (**b**) genomes. The red arrows represent the positions that paired ends of a sweetpotato BAC matched on the chromosomes of *S. lycopersicum* (**a**) and *V. vinifera* (**b**); the purple arrows represent the genes distributed on the chromosomes of *S. lycopersicum* (**a**) and *V. vinifera* (**b**); the arrows (red and purple) in right direction represent matching on sense strand, the arrows (red and purple) in left direction represent matching on antisense strand

According to the method of Rampant et al. [32], the matches are classified into 2 categories: ‘single end’ (SE) and ‘paired end’ (PE). The PE category is subdivided into ‘non-colocalized’ and ‘colocalized’ and the latter includes ‘collinear’, ‘rearranged’ and ‘gapped’. A total of 491 BESs (477 SEs and 14 PEs) were matched to the genome sequences of *S. lycopersicum* (Fig. 7). Twelve of the 14 PEs were ‘non-colocalized’, and 2 (R358H9 and F358H9) mapped to the *S. lycopersicum* chromosome 3 within ~69 kb apart fell into the ‘collinear’ category, suggesting the presence of one putative microsyntenic region between sweetpotato and *S. lycopersicum* (Fig. 7). R358H9 began at position 65,487,266 bp and F358H9 terminated at position 65,556,186 bp (Fig. 8). This region contained 15 genes, seven on sense strand and eight on antisense strand (Fig. 8). Comparative mapping between sweetpotato and *V. vinifera* revealed 272 matches, including 268 SEs, 2 ‘non-colocalized’ and 2 ‘collinear’ (R157E19 and F157E19). R157E19 and F157E19 formed ‘collinear’ alignment on the *V. vinifera* chromosome 3 within ~310 kb apart, the reverse end beginning at position 1,882,624 bp and the forward end terminating at position 2,193,110 bp. This region encompassed 32 genes, 9 on sense strand and 23 on antisense strand (Fig. 8). In addition, 221 matches (217 SEs, 2 ‘non-colocalized’ and 2 ‘rearranged’) to the *T. cacao* genome and 99 matches (97 SEs and 2 ‘gapped’) to the *A. thaliana* genome were identified (Fig. 7). In the perspective of the whole genome, the matches dispersed on all chromosomes of *S. lycopersicum*, *V. vinifera*, *T. cacao* and *A. thaliana* and 36 of them were found in all of the four genomes (Fig. 7, Additional file 15: Table S13).

## Discussion

Sweetpotato is a highly heterozygous autohexaploid and its genomic BAC library has not been reported to date. In the present study, the BAC library of sweetpotato was successfully constructed using the elite line Xu 781 of this crop. The BAC library consisted of 240,384 clones, the majority (75.42%) of which had insert sizes of > 90 kb, with an average insert size of 101 kb, similar to the results reports in several plant species such as sugar beet [33], peanut [34], narrow-leafed lupin [35] and passion fruit [29]. Thus, this library has a reasonably large average insert size. The BAC library provided a 7.93–10.82 × coverage of the sweetpotato genome, with more than 99% probability of isolating any single-copy DNA sequence from the library. The coverage of this library is greater than those of peanut [34] and passion fruit [29], and comparable to those of sugar beet [33] and narrow-leafed lupin [35]. Additionally, the present library was constructed from a partial digestion of genomic DNA using only one restriction enzyme (*Hind*III), which was also used to construct the libraries of sugar beet (*Bam*HI) [33], peanut (*Hind*III) [34], narrow-leafed lupin (*Bam*HI) [35] and passion fruit (*Hind*III) [29]. This might lead to preferential cloning owing to the uneven distribution of restriction sites throughout the genome, which could be minimized by developing more clones for the library using one or two new restriction enzymes in the future [33, 36]. This is the first large-insert BAC library for sweetpotato and a valuable tool for future sequencing and genome studies.

The present study provides a first overview of the structure and composition of the sweetpotato genome through the analysis of 11,542 high quality BESs. The GC content of the sweetpotato genome was 38.18%, comparable to the 38.45% of the chloroplast genome of this species and the 35.6–36.0% of *I. trifida* (2×) [6, 14]. Therefore, these results suggest that the genomes of sweetpotato and its wild relatives are all AT-rich.

Repetitive DNA, as a significant portion of most eukaryotic genomes, plays important roles during polyploidization and post-polyploidization changes [37, 38]. The *I. trifida* (2×) genome is estimated to be composed of 42.3–47.7% repeats [6]. Repetitive sequences account for at least 62.2% of the assembled potato genome [39] and this proportion reaches approximately 80% in wheat [40]. The present results revealed that the repetitive DNA in the sweetpotato genome was approximately 30.48%, with 12.17% homologous to known repeats and 18.31% specific to sweetpotato. The proportion of repeats in sweetpotato might be underestimated, as reported in many plant species such as *S. tuberosum*, *S. lycopersicum* and *S. maritime* [26, 36].

It is known that transposable elements have important consequences on genome structure and functions [41]. The present study indicated that Class I retrotransposons (8.51%) were significantly predominant compared to Class II DNA transposons (1.42%) in the sweetpotato genome, as in other plant genomes [39, 40, 42]. Further analysis revealed that the percentage of Class I retrotransposons was much larger in sweetpotato than in *I. trifida* (4.8–5.2%), but the proportion of Class II DNA transposons in sweetpotato was comparable to that in *I. trifida* (1.4–1.5%). Ty1-copia and Ty3-gypsy retrotransposons are two main types of LTRs, playing important roles in maintaining chromatin structures and centromere functions and regulating gene expression in the host genomes [43]. The ratio of Ty3-Gypsy:Ty1-Copia in sweetpotato was approximately 1.15:1, indicating that the contributions of Ty3-gypsy and Ty1-copia to the sweetpotato genome were approximately equal. This ratio is similar to those of *Passiflora edulis* (1:1) [29], *M. guttatus* (1:1), *Prunus persica* (1:1.18) and *P. edulis* (1.06:1), but lower than those of *S. lycopersicum* (2.45:1), *S. tuberosum* (2.48:1), *V. vinifera* (1:3) and *A. thaliana* (2.94:1) [28]. In addition, the novel repetitive elements were found in the sweetpotato genome. They were classified as DNA transposons, LTRs, LINEs, SINEs and unknown. These repeats should be further used to study genome structure and functions of sweetpotato.

The proportion of the sweetpotato BESs with potential coding regions was moderate compared to the assessment of coding regions in many BES-based studies [26, 27, 44–46]. The cumulative coding region length was 760,248 bp, representing 10.00% of the total sweetpotato BESs length. Based on matches to the protein databases of *S. lycopersicum* and *V. vinifera*, the total coding sequences of the sweetpotato genome were predicted to be 217.40–296.97 Mb and the gene content was estimated as 146,792–215,351. Thus, the gene content is much higher in sweetpotato than in diploid *I. trifida* (62,407–109,449) [6]. The large gene content might be caused by highly heterozygosity and the polyploidy nature of sweetpotato [5].

SSR markers are widely used for genome analysis and map comparison and consensus due to their abundance, functionality, high polymorphism and excellent reproducibility [47]. BESs have been proven to be valuable sources of SSRs [48, 49]. In our study, a total of 3,846 SSRs were identified from the 2,698 sweetpotato BESs. The average density of SSRs was one SSR per 1.93 kb in sweetpotato, close to those in *Carica papaya* (one SSR per 1.72 kb), *A. thaliana* (one SSR per 1.82 kb), *Amborella trichopoda* (one SSR per 1.88kb) and *Citrus clementine* (one SSR per 1.99 kb), and higher than those in the other surveyed species (Additional file 9: Table S9). Our results also showed that potential SSRs were more numerous in the sweetpotato BESs than in the ESTs, as reported in walnut and coffee [19, 28]. Furthermore, the amplification results indicated that the 60.07% of primer pairs designed from the chosen SSRs exhibited good polymorphism among 20 sweetpotato accessions with differences in yield, quality and diseases resistance (Additional file 11: Figure S1), which was higher than 32.26% of primer pairs from EST-SSRs reported by Wang et al. [50]. The present BES-SSRs also showed good polymorphism among 168 F_1_ individuals of Xushu 18 × Xu 781 (Additional file 12: Figure S2). Therefore, these BES-SSRs can be used to identify germplasm, assess genetic diversity, construct genetic linkage maps and develop molecular markers for agronomically important traits in sweetpotato.

Cytogenetic and molecular genetic evidences indicate that *I. trifida* (2×) is the most likely diploid ancestor of the hexaploid sweetpotato [51–54]. In the present study, 97.29% and 98.08% of the sweetpotato BESs were matched to the genome sequences of Mx23Hm and 0431–1 of *I. trifida* (2×), covering 82.02% and 81.03% of the total BESs length, respectively. These results provide the genomic evidence for the highly close relationship between sweetpotato and *I. trifida* (2×). Moreover, the BAC clones, with both ends aligned on the same contigs of *I. trifida* (2×) in the correct orientation within 15–350 kb apart, were also identified and might be used in comparative genomics study between sweetpotato and *I. trifida.* Comparative mapping between both species can not be performed due to the fact that *I. trifida* genome is still in the early stages of its assembly and annotation [6].

Well-sequenced species with the highest number of top hits are commonly used as reference genomes for the BAC-end analysis of target species [26–29, 32]. Our study revealed that more sweetpotato BESs were matched to *S. lycopersicum* than *V. vinifera*, *T. cacao* and *A. thaliana* (Fig. 7). It is consistent with the fact that sweetpotato and *S. lycopersicum*, belonging to Solanales, diverged from a common ancestor approximately 82–86 million years ago, while the divergence between sweetpotato and *V. vinifera*, *T. cacao* and *A. thaliana* is estimated to be approximately 123–125 million years ago [55]. More sweetpotato BESs were matched to the genome of *V. vinifera* than those of *T. cacao* and *A. thaliana*, which might be because *V. vinifera* did not undergo recent genome duplication [56]. Similarly, the limited number of sweetpotato BESs matched to the *A. thaliana* genome might be because the *A. thaliana* genome suffered many gene losses since its two whole-genome duplications [56]. These findings provide an interesting starting point for comparative genomics and evolution studies of sweetpotato.

## Conclusions

The first genomic BAC library for sweetpotato has been successfully constructed. It has a highly redundant genome coverage (7.93–10.82 ×), and contains large inserts (101 kb) and a very low frequency of clones derived from the mitochondrial genome and chloroplast genome. The high quality BESs provide first insights into sweetpotato genome composition, including GC content, transposable elements and protein coding regions, and have significant hits to the genome sequences of *I. trifida* and more matches to the whole-genome sequences of *Solanum lycopersicum*. SSRs identified from the BESs show good polymorphism in sweetpotato. These resources as a robust platform will be used in high-resolution mapping, gene cloning, assembly of genome sequences, comparative genomics and evolution for sweetpotato.

## Methods

### Plant materials

The sweetpotato line Xu 781 was used to construct a BAC Library. Xu 781 was selected from bulked seeds of JPKY0-015 in an open-pollinated poly-cross and conserved at our laboratory [57]. It has high dry-matter content and stem nematode resistance and is extensively used as a parent in sweetpotato breeding programs in China. After plants were grown in the dark for 7 days, their young leaves were collected and rapidly frozen by submersion in liquid nitrogen, followed by temporarily storing at −80 °C for DNA isolation.

### BAC library construction

About 20 g leaves of Xu 781 were ground into powder in a mortar containing liquid nitrogen. The isolation of high molecular weight (HMW) DNA was conducted according the procedure of Zhang et al. [58]. Four DNA plugs were partially digested for 8 min at 37 °C with 0, 10, 20, and 30 units of *Hind*III (New England Biolabs, Beijing, China), respectively, to determine optimal partial digestion conditions. The digested plugs were separated by two rounds of pulsed field gel electrophoresis (PFGE) at 6 V/cm with a 5–15 s switch time for 16 h at 14 °C to elute DNA fragments ranging from 100 kb to 300 kb in size. The target DNA fragments were ligated into the CopyControl™pCC1BAC™ Vector (Epicentre Biotechnologies, Madison, WI, United States) at 16 °C overnight. Two μl of the ligation product were used to transform 20 μl of *E. coli* EPI300 cells (Epicentre Biotechnologies, Madison, WI, United States) by electroporation at 14 KV/cm. The cells were then cultured on Luria Broth (LB) medium containing 12.5 μg/ml chloramphenicol, 60 μg/ml 5-bromo-4-chloro-3-indolyl-β-d-galactopyranoside (X-Gal) and 15 μg/ml isopropyl β-D-1-thiogalactopyranoside (IPTG) for 24 h at 37 °C. Recombinant clones were picked manually, arrayed into 384-well plates containing 80 μl of LB freezing medium with 12.5 μg/ml chloramphenicol, incubated at 37 °C overnight, and then stored at −80 °C.

### BAC library characterization

A set of clones randomly selected from the BAC library were cultured in 4 ml LB medium containing 12.5 μg/ml chloramphenicol on a reciprocal shaker (200 strokes/min) at 37 °C overnight. Plasmid DNA (~1 μg) of the BAC clones was isolated according to standstard alkaline-lysis method [59], and digested with 5 U of restriction enzyme *Not*I (New England Biolabs, Beijing, China). The digested products were separated by PFGE on an 1% agarose gel at 6 V/cm with a 5–15 s switch time for 16 h at 14 °C, and the electrophoresis results were detected by ethidium bromide (EB) staining. The insert size of each clone was determined by comparing the bands to MidRange PFG Marker I and MidRange PFG Marker II (New England Biolabs, Beijing, China). The genome coverage of the BAC library and the probability of isolating any single-copy DNA sequence from the library were estimated according to the method of Clarke and Carbon [60].

A set of the BAC clones were randomly selected as templates for PCR to estimate the level of contamination by organellar DNA. Primers for 2 mitochondrial genes (*matR*, GenBank: GU351235.1; *nad5*, GenBank: GU351439.1) and 2 chloroplast genes (*psaA*, GenBank: KP212149.1; *psbA*, GenBank: KP212149.1) (Additional file 16: Table S14) were designed by Primer3 [61, 62]. The reaction mixture consisted of 2 μl 10 × PCR buffer, 1.6 μl 2.5 mM dNTPs, 1 μl of each primer (10 μM), 1 μl (~50 ng) BAC DNA, 0.2 μl (1 U) EasyTaq® DNA Polymerase (TransGen Biotech, Beijing, China) and 14.2 μl double-distilled water. PCR amplifications were programmed as follows: 94 °C for 5min, followed by 35 cycles of 94 °C for 30 s, 60 °C for 30 s, 72 °C for 30 s, and then a final 10 min extension at 72 °C. PCR products were gently mixed with 4 μl 6 × DNA loading buffer (TransGen Biotech, Beijing, China), and then 5 μl of the mixture were loaded onto a 1% agarose gel and separated by electrophoresis at 6 V/cm for 21 min at room temperature. Electrophoresis results were detected by GoldView (YeaSen, Beijing, China) staining.

To identify the BAC clones containing sweetpotato genes of interest, the library was screened using the primers designed from cDNA sequences of sweetpotato *myo*-inositol-1-phosphate synthase gene (*IbMIPS1*) and polyphenol oxidase gene (*IbPPOS*) (Additional file 16: Table S14) as described by Farrar et al. [63]. The BAC clones with target sequence were identified by PCR amplifications as described above.

### BAC-end sequencing

A set of the BAC clones were randomly selected and incubated in 96-well deep-well plates containing 1.5 ml of 2× LB medium with 12.5 μg/ml chloramphenicol for 20 h on a reciprocal shaker (200 strokes/min) at 37 °C. BAC DNA was isolated and purified using standstard alkaline-lysis method [59]. BAC-end sequencing was performed in the forward and reverse directions using BigDye Terminator V 1.1 and ABI PRISM 3730 DNA Analyzer technologies (Applied Biosystems, Life Technologies Corporation, Foster, CA, United States) at Corporation of Beijing Genomics Institute (BGI), China. Base calling of ABI trace files was conducted using Phred software [64]. The bases with Phred quality score < 20 were trimmed, and the vector sequences were subsequently removed using CROSS_MATCH [65]. After filtering out the sequences with a length shorter than 100 bp, the organellar DNA sequences were removed by comparing the BESs with the *Arabidopsis* mitochondrial genome (GenBank: NC_001284.2) or chloroplast genome (GenBank: NC_000932.1) using BLASTN with an E-value cutoff of 1e-15.

### Repetitive sequence identification

The repetitive sequences in the sweetpotato BESs were identified and masked by searches for similarity to sequences in the eukaryote section of the RepBase repeat database (ver. 2013042) with CROSS_MATCH and RepeatMasker [66]. The masked sequences were further scanned to identify de novo repeats using RepeatModeler [67]. The repeats were compared against the NCBI NR database using BLASTX with an E-value cutoff of 1e-06, and then the repeats containing hits to transposon related proteins were eliminated from the list of novel repeats. The novel repeats were classified using TEclass [68], and then were used as a custom library for RepeatMasker to further mask repetitive sequences in the sweetpotato BESs.

### Function annotation

The sweetpotato BESs without known and novel repeats were analyzed for protein coding regions by comparing with the sweetpotato ESTs downloaded from NCBI GenBank and derived from our in-house transcriptome data (unpublished), NCBI-EST database and *I. trifida* (Mx23Hm and 0431–1) CDS databases [6] using BLASTN with an E-value cutoff of 1e-10. Further analysis for these BESs was conducted by comparing with NCBI NR protein database, *S. lycopersicum* protein database [69] and *V. vinifera* protein database [70] using BLASTX with an E-value cutoff of 1e-06. The total match lengths of these searches were calculated to estimate the protein coding regions and gene content in sweetpotato. BLAST2GO software was used for GO functional annotation and classification [71].

### SSR detection

BES-SSRs types (mononucleotide to hexanucleotide) were identified using MISA [72]. The distribution and frequency of SSRs in the sweetpotato BESs were compared with those in sweetpotato ESTs downloaded from NCBI GenBank and derived from our in-house transcriptome data (unpublished) and in other species BESs downloaded from NCBI [73]. All of the analyses required a minimum length of 20 bp for mononucleotide repeats and at least 15 bp for dinucleotide-to-hexanucleotide repeats, and two or more SSRs separated by ≤ 100 bp were considered as a compound SSR.

Twenty sweetpotato accessions, Zhenghong 22, Yushu 10, Jishu 10, Jishu 98, Xu 43–14, Lushu 3, Shangshu 19, Lizixiang, Xu 781, Xushu 18, Dayebai, Norin 1, Tielizi, Shagenshao, Beijing 553, Nancy Hall, Datouhuang, Jinguahuang, Baidumian and Mengziyanghong (Additional file 17: Table S15) and 168 F_1_ individuals of Xu 781 and Xushu 18 were used to assess polymorphisms of the developed SSRs. PCR amplifications were performed according to the method of Zhao et al. [57]. PCR products were separated by electrophoresis on the 5% (w/v) denaturing polyacrylamide gels and 3% (w/v) agarose gels, respectively.

### Comparative genome analysis

The sweetpotato BESs were compared with the genome sequences of Mx23Hm and 0431–1 of *I. trifida* [6] using BLASTN with an E-value cutoff of 1e-06. The BLASTN results were further filtered based on criteria: identity ≥ 70%, alignment length ≥ 50 bp [26]. Furthermore, these BESs were compared with the sequenced *S. lycopersicum*, *V. vinifera*, *T. cacao* and *A. thaliana* genomes downloaded from NCBI [73] to identify the potential microsynteny using BLASTN with an E-value cutoff of 1e-06. The BLASTN results were further filtered as mentioned above. The matches were classified according to the method of Rampant et al. [32]. The best matches between sweetpotato BESs and *S. lycopersicum*, *V. vinifera*, *T. cacao* and *A. thaliana* genomes were used for synteny visualization using the Circos program [74].

## Additional files

Additional file 1: Table S1. The BLASTN searching results of 4428 BESs with sweetpotato EST database. (XLS 635 kb)
Additional file 2: Table S2. The BLASTN searching results of 4428 BESs with NCBI EST database. (XLS 288 kb)
Additional file 3: Table S3. The BLASTN searching results of 4428 BESs with sweetpotato EST and NCBI EST database. (XLS 646 kb)
Additional file 4: Table S4. The BLASTX searching results of 3422 BESs with NCBI NR database. (XLS 397 kb)
Additional file 5: Table S5. The BLASTX searching results of 4428 BESs with protein databases of *S. lycopersicum*. (XLS 416 kb)
Additional file 6: Table S6. The BLASTX searching results of 4428 BESs with protein databases of *V. vinifera*. (XLS 400 kb)
Additional file 7: Table S7. The BLASTN searching results of 11542 BESs with Mx23Hm database. (XLS 479 kb)
Additional file 8: Table S8. The BLASTN searching results of 11542 BESs with 0431–1 database. (XLS 466 kb)
Additional file 9: Table S9. Distribution and frequency of SSRs detected in different plant species. (XLS 28 kb)
Additional file 10: Table S10. List of 288 SSR pairs developed from the sweetpotato BESs for assessing their polymorphisms. (XLS 75 kb)
Additional file 11: Figure S1. PCR amplification of the 20 sweetpotato accessions using the 6 BESs-SSR pairs. (A) Denaturing polyacrylamide gels. M1: GeneRuler™ 50bp DNA Ladder; M2: GeneRuler™ 100bp DNA Ladder. (B) Agarose gels. M: BL 2000 DNA Marker. (JPG 632 kb)
Additional file 12: Figure S2. PCR amplification of Xu 781 (I) and Xushu 18 (II) and their 168 F_1_ individuals with BES_SSR_267. M: BL 2000 DNA Marker; Lanes 1 to 168: 168 individuals. (TIF 1647 kb)
Additional file 13: Table S11. Paired BESs aligned on the same contigs of Mx23Hm in the correct orientation. (XLS 106 kb)
Additional file 14: Table S12. Paired BESs aligned on the same contigs of 0431–1 in the correct orientation. (XLS 50 kb)
Additional file 15: Table S13. The 36 BESs matched to all the four sequenced genomes. (XLS 30 kb)
Additional file 16: Table S14. Primers used for screening BAC library. (XLS 26 kb)
Additional file 17: Table S15. Sweetpotato accessions used for BES-SSRs validation and evaluation. (XLSX 11 kb)
Additional file 18: Text S1. The list of NCBI Genbank accession numbers for sweetpotato BESs. (TXT 298 kb)

## Abbreviations

BAC: Bacterial artificial chromosome
BESs: BAC-end sequences
ESTs: Expressed sequence tags
GO: Gene ontology
HMW: High molecular weight
*IbMIPS1*: *Ipomoea batatas* myo-inositol-1-phosphate synthase gene
*IbPPOS*: *Ipomoea batatas* polyphenol oxidase
IPTG: Isopropyl β-D-1-thiogalactopyranoside
NCBI: National center for biotechnology information
NR: Non-redundant protein (NR) database
PCR: Polymerase chain reaction
PFGE: Pulsed field gel electrophoresis
SSR: Simple sequences repeat
X-Gal: 5-bromo-4-chloro-3-indolyl-β-d-galactopyranoside
