# Supplementary figures and images for: A genome-wide BAC-end sequence survey provides first insights into sweetpotato (Ipomoea batatas (L.) Lam.) genome composition
Source: BMC Genomics. 2016 Nov 21;17:945. doi: 10.1186/s12864-016-3302-1 (PMC5117676; doi:10.1186/s12864-016-3302-1)

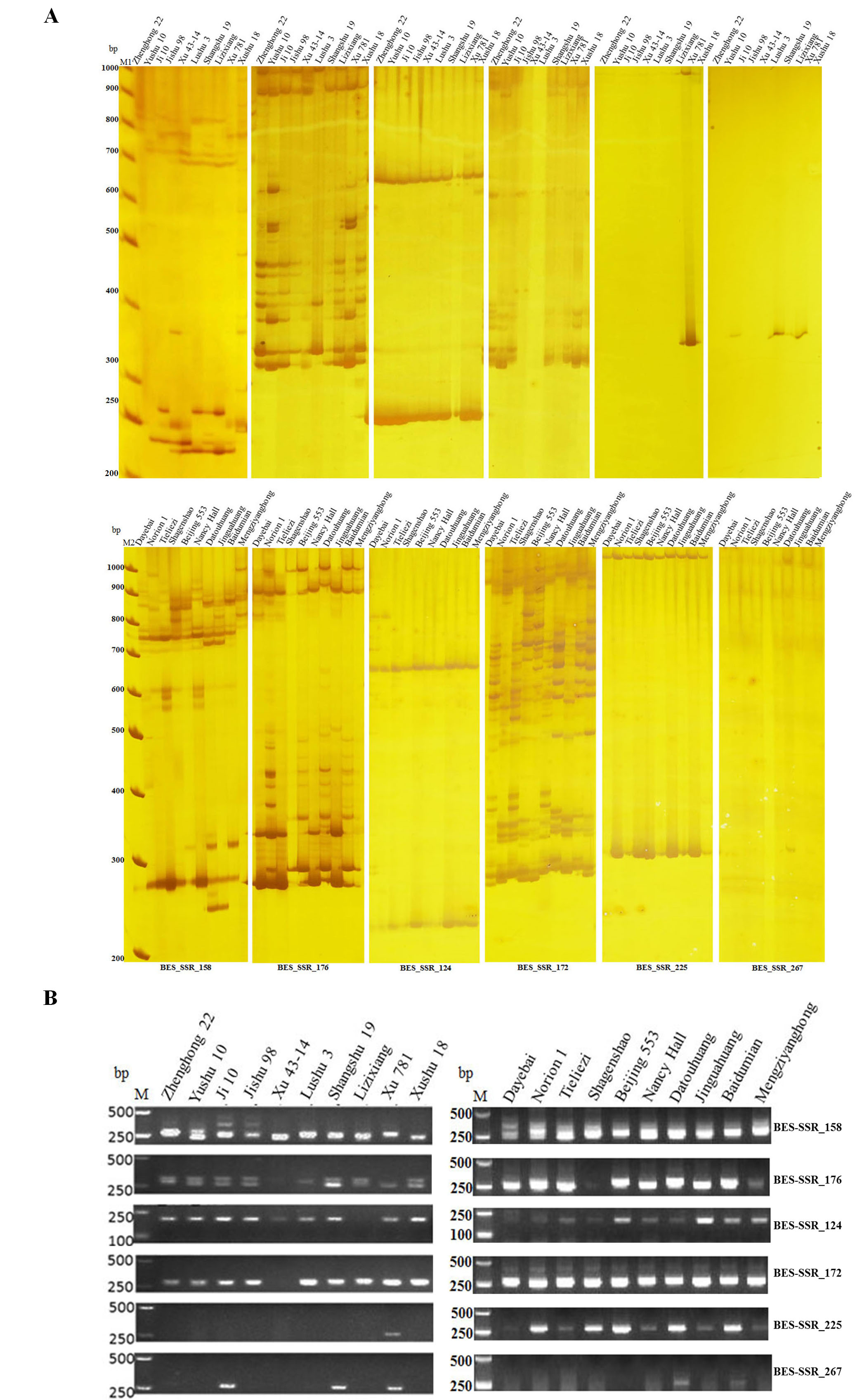

Supplement: Additional file 11: Figure S1. — PCR amplification of the 20 sweetpotato accessions using the 6 BESs-SSR pairs. (A) Denaturing polyacrylamide gels. M1: GeneRuler™ 50bp DNA Ladder; M2: GeneRuler™ 100bp DNA Ladder. (B) Agarose gels. M: BL 2000 DNA Marker. (JPG 632 kb) [file 12864_2016_3302_MOESM11_ESM.jpg]

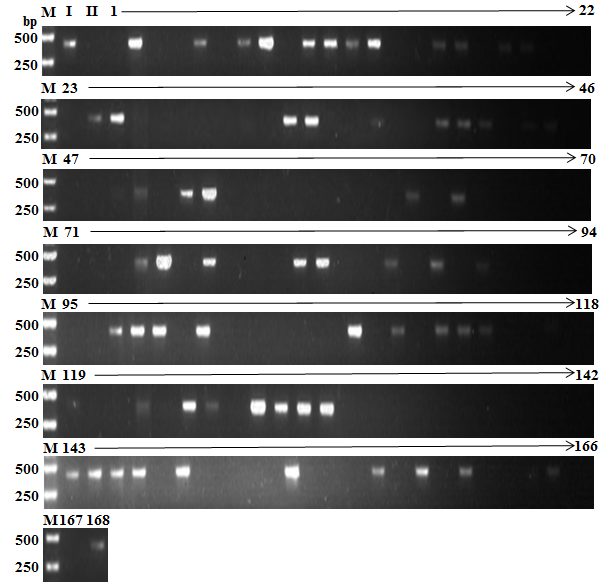

Supplement: Additional file 12: Figure S2. — PCR amplification of Xu 781 (I) and Xushu 18 (II) and their 168 F1 individuals with BES_SSR_267. M: BL 2000 DNA Marker; Lanes 1 to 168: 168 individuals. (TIF 1647 kb) [file 12864_2016_3302_MOESM12_ESM.tif]
